# Health and Artistry

**Published:** 1920-10-30

**Authors:** 


*To the Editor of* The Hospital.

### Health and Artistry.

*To the Editor of* The Hospital.

Sir,—Writing as an unqualified member of the general public, but keenly interested in certain aspects of our health problems, I have often felt the serious need for expert pronouncement concerning the relation between an overstrung nervous system, the want of balance in the individual make-up, and the attainment of superlative artistry.

I hesitate to think that the general artistic perceptions form fit subject for regular lifelong exploitation as a profession or means of livelihood. Were I the parent of a child "gifted" in this way, I should reason thus. A steady outlook and temperate views constitute the most serviceable base of practical living. Therefore, instead of sedulously tending this endowment to glorious fruition, I should be inclined to have the lad carefully examined by a medical man, to ascertain if there were any predisposition to disease, serious nervous disorder, or other source of irritation and restless activity. "Temperament" seems to me a thing to be carefully avoided, and the incessant temptation to artificial over-stimulus in pursuing the artistic career would certainly mark it as undesirable for a parent wishful to develop in his boy the steady-going, good-humoured, practical way of life which instinctively shuns extremes. Attention to regular sleep, nutritious but unstimulating diet, plenty of unstrained physical occupation, much outdoor life, little book work or reading, enough to make life cheerful, but not to encourage "temperament," and an environment of practical, good-humoured folk, would be advisable in these cases.

Let me, however, make this quite clear. The perception of what is fine and beautiful in life, in pictorial art, music, and verbal expression is a quality grievously lacking in the average man. If we had no transcendent artist, living an impossibly overstrung life, and instead every man could draw very well, possessed a trained singing voice, and used words with fair precision, the atmosphere of social life would be infinitely more favourable to the dissemination of generous and kindly ideals and the elimination of the rude and crude competitive element.

To sum up, the office of the æsthetic faculties seems to be the effecting of modulating our frame of mind, tending to sociable, pleasant, and intelligent recreation, which must be earned by balancing exertion in more serious and practical activities.—I am, Sir, yours truly, "A."

[Some years ago the late Dr. James Mercier contributed to these columns a series of articles on Human Temperament, which were subsequently published in book form by the Scientific Press. Dr. Mercier discriminated strongly between what he called the "Artistic Temperament" and the "Temperament of the Artist." We think his views would interest our correspondent, and we advise him to get hold of a copy of Dr. Mercier's booklet.—Ed. The Hospital.]

